# Use of Facial Morphology to Determine Nutritional Status in Older Adults: Opportunities and Challenges

**DOI:** 10.2196/33478

**Published:** 2022-07-18

**Authors:** Wesley Tay, Rina Quek, Bhupinder Kaur, Joseph Lim, Christiani Jeyakumar Henry

**Affiliations:** 1 Clinical Nutrition Research Centre Singapore Institute of Food and Biotechnology Innovation Agency for Science, Technology and Research Singapore Singapore; 2 Department of Biochemistry Yong Loo Lin School of Medicine National University of Singapore Singapore Singapore

**Keywords:** malnutrition, facial recognition, facial morphology, telemonitoring, 3D scans, digital health, digital nutrition, public health nutrition, mobile phone

## Abstract

Undiagnosed malnutrition is a significant problem in high-income countries, which can reduce the quality of life of many individuals, particularly of older adults. Moreover, it can also inflate the costs of existing health care systems because of the many metabolic complications that it can cause. The current methods for assessing malnutrition can be cumbersome. A trained practitioner must be present to conduct an assessment, or patients must travel to facilities with specialized equipment to obtain their measurements. Therefore, digital health care is a possible way of closing this gap as it is rapidly gaining traction as a scalable means of improving efficiency in the health care system. It allows for the remote monitoring of nutritional status without requiring the physical presence of practitioners or the use of advanced medical equipment. As such, there is an increasing interest in expanding the range of digital applications to facilitate remote monitoring and management of health issues. In this study, we discuss the feasibility of a novel digital remote method for diagnosing malnutrition using facial morphometrics. Many malnutrition screening assessments include subjective assessments of the head and the face. Facial appearance is often used by clinicians as the first point of qualitative indication of health status. Hence, there may be merit in quantifying these subtle but observable changes using facial morphometrics. Modern advancements in artificial intelligence, data science, sensors, and computing technologies allow facial features to be accurately digitized, which could potentially allow these previously intuitive assessments to be quantified. This study aims to stimulate further discussion and discourse on how this emerging technology can be used to provide real-time access to nutritional status. The use of facial morphometrics extends the use of currently available technology and may provide a scalable, easily deployable solution for nutritional status to be monitored in real time. This will enable clinicians and dietitians to keep track of patients remotely and provide the necessary intervention measures as required, as well as providing health care institutions and policy makers with essential information that can be used to inform and enable targeted public health approaches within affected populations.

## Introduction

### Background

Malnutrition in older adults in many high-income countries is an unrecognized condition that is increasing in prevalence as the population ages [1]. The impact of malnutrition on morbidity and mortality in older adults is a problem that necessitates urgent attention. The aging process brings about biological, physiological, social, and psychological changes, coupled with a higher morbidity prevalence, which can negatively affect nutritional status [2].

Malnutrition in older adults, also coined as *anorexia of aging*, has severe implications, resulting in a significantly poor quality of life [3,4]. The loss of swallowing function, polypharmacy, cachexia, and lack of social support can exacerbate malnutrition [5,6]. Malnutrition occurs because of insufficient food intake, the inability of the body to absorb nutrients, or because of the presence of catabolic diseases such as chronic obstructive pulmonary disease or cancer. This can cause a reduction in fat and muscle mass, further leading to impaired physical and cognitive function [7]. Malnutrition can occur gradually over significant periods without being detected, as the symptoms may not be critical to warrant immediate medical attention. However, the severity of malnutrition may be significantly elevated if it is associated with a disease or inflammation. In geriatric patients with diseases, it is evident that a reduction in food intake causes increased muscle breakdown because of increased resting energy expenditure [8].

Malnutrition has been found to lead to sarcopenia, and sarcopenia may aggravate malnutrition, leading to the aggravation of morbidity and mortality [9,10]. Sarcopenia is defined as an age-related loss of muscle mass, where muscle mass is lost involuntarily over time, leading to a decrease in muscle protein synthesis, strength, and function [11]. In a recent review, both malnutrition and sarcopenia were examined concomitantly, and the findings for the management and interventions of these 2 conditions were found to overlap considerably [12]. Hence, the newly proposed clinical syndrome, called malnutrition-sarcopenia syndrome, should be assessed simultaneously to improve health outcomes [13]. Older adults have an increased risk of malnutrition compared with other adult populations, and it has been estimated that 2% to 16% of community-dwelling older adults are deficient in their protein and energy needs [14]. The effects of poor skeletal muscle mass (SMM) can be detrimental as they reduce the prognosis of many diseases, including diabetes, heart disease, chronic kidney disease, sepsis, and cancer [15-20]. In a community-based, cross-sectional study conducted on 722 stroke- and dementia-free participants aged 50 to 75 years, an increase in SMM was observed to protect against ischemic stroke, especially in men [21]. In another study in which the prevalence of sarcopenia in 414 patients with diabetes and 396 control participants was examined, type 2 diabetes was independently associated with sarcopenia, with a 15.7% prevalence in patients with diabetes compared with 6.9% in the control group [17].

Apart from physiological and metabolic implications, malnutrition and sarcopenia in older adults have been widely established to have financial implications and are burdens to health care resources [22]. Patients who are malnourished are associated with more frequent hospitalizations, increased length of hospital stays, and increased readmissions and hospitalization costs [23]. Approximately 40% of patients are malnourished upon hospital admission, and depending on the severity, they are faced with approximately 31% to 38% increase in hospital costs [24].

However, it is difficult to detect subclinical malnutrition because of the absence of clear indicators. This could lead to a lack of identifying poor nutritional practices among older adults, as malnutrition does not often present with immediate problems. Therefore, better screening options for the diagnosis of subclinical malnutrition must be recognized and identified [25,26]. This could potentially ease the burden on the health care system, reduce the cost of resources, improve the quality of patients’ lives, and extend the healthy living years of older adults.

It is imperative that malnutrition and the prognostic value of treatment options to manage malnutrition be identified. The face is often used by health practitioners to make qualitative assessments of health and nutritional status. Modern advancements in sensor and computing technologies enable features in the face to be accurately digitized and could potentially allow these previously intuitive assessments to be quantified. In this paper, we discuss the feasibility of a novel digital remote method for diagnosing malnutrition using facial morphometrics.

### Current Methods of Diagnosing Malnutrition

Until 2016, there were no universally accepted screening tools or criteria used as the gold standard to identify malnutrition risk [27]. The Global Leadership Initiative on Malnutrition assembled a set of criteria for identifying and diagnosing malnutrition in older adults in hospitals [27]. This initiative comprises a 2-step method in which a validated screening tool is used to identify patients who are at risk of malnutrition. This is followed by an assessment to diagnose and grade the severity of malnutrition. The Global Leadership Initiative on Malnutrition grouped the diagnosis of malnutrition into two distinct categorical criteria: phenotypic (which included weight loss, low BMI, and reduced SMM) and the other etiologic criteria comprising reduced food intake, disease burden, or inflammation [27].

One of the most widely used traditional methods for assessing malnutrition is the subjective global assessment tool. It is an integrated tool that assesses nutritional status based on patients’ past records and physical examinations, and subsequently classifies patients into three categories: well-nourished, moderately malnourished, or severely malnourished [28]. Anthropometric measurements such as midarm circumference and triceps skinfold thickness have been used as basic methods of correlating the percentage of body fat and lean muscle mass [29]. In addition to the subjective global assessment, bioelectrical impedance analysis and ultrasound techniques have been recognized as accurate methods for measuring lean body tissue, which is a key indicator of nutritional status [30]. Other established methods used to assess SMM include scans using computed tomography (CT), dual-energy x-ray absorptiometry, and magnetic resonance imaging (MRI), which are costly and time consuming [31,32]. Analytic morphomics is an emerging field that uses cross-sectional images to provide a global assessment of a patient beyond the localized specific pathology of interest (Englesbe et al [33]). Through the application of CT-based analytic morphomics, Lee et al [31] developed the Morphomic Malnutrition Score as a means of providing standardization and objective measurements for conventional diagnostics. This method provides an in-depth assessment to distinguish between healthy individuals and individuals who are severely malnourished. Although these methods are highly accurate, conducting them can be cumbersome and resource intensive, as they require a trained practitioner to be present to conduct the assessment, and patients need to travel to specialized facilities to obtain their measurements. In cases where older adults do not frequent clinics or hospitals, malnutrition and sarcopenia could be left undetected for extended periods or until the onset of a disease, leading to several negative implications. If malnutrition monitoring and assessments could be delivered or enhanced through the use of readily accessible and cost-effective imaging technologies, this could greatly reduce the burden on rapidly inflating health care costs. More importantly, the implementation of such methods would significantly improve access to and quality of care for older adult patients who are malnourished.

## Telemonitoring and Digital Health Care Systems

### Overview

Digital health care is one such approach that allows for malnutrition and nutritional status to be monitored remotely without requiring the physical presence of practitioners or advanced equipment. As we enter the Fourth Industrial Revolution, the widespread availability and improvements in data science and infocommunication (integration of information and telecommunication technology sectors) technologies have rapidly expanded the vast range of available services and applications in the health care sector [34-39]. Digital health care, in its many forms, is rapidly gaining traction as a scalable means of improving efficiency in the health care system, allowing health care institutions to extend their reach and increase the number of touchpoints they have with patients. Increased levels of connectivity offered by these technologies are changing the ways in which the health care system is structured and administered.

Telemonitoring is the real-time monitoring of patients using mobile technologies to conduct routine medical tests and communicate results to health care workers for evaluation [38]. This capitalizes on the recent progress in Internet of Medical Things devices and widespread internet access, which can range from data gathered from a single device to an entire network of personal digital devices, connected medical devices, implants, and other sensors [38]. The integration of these sensors and collection of accurate real-time data can support clinical decisions by rapidly communicating changes in physiological or biochemical states, improving the overall quality of health care services and leading to more effective management of chronic diseases [34,37,38,40-46]. Digital applications allow conditions to be managed at home or within local communities, offering patients greater levels of independence and autonomy, reducing travel time, and ensuring better equity for patients in rural areas [34,38]. As such, many studies have shown high levels of satisfaction, empowerment, and reassurance expressed by both providers and end users of telemonitoring solutions [34,37,45,47].

Telemonitoring is also considered an efficient use of health care resources [34,37,38,40-44,46,48]. Many conventional tests that require the presence of a health care worker can now be conducted automatically with the use of specialized, portable equipment, reducing the number of face-to-face interactions required of health care workers [34,38]. Patients are also not required to be physically present at health care institutions. The reduction in traffic and congestion allows for the prioritization of more serious cases that require immediate care, a benefit most notably realized during the COVID-19 pandemic [34,49,50]. The improved management of chronic diseases also reduces the number of hospitalization events and the length of hospital stays for many conditions and, consequently, reduces overall health care expenditure.

Given the many benefits that telemonitoring has to offer, it is critical that new applications are developed to broaden the scope of conditions to which telemonitoring can be applied. In both high- and low-income nations, smartphones have been used as platforms for several aspects of life, such as identity verification and personal banking, and are increasingly being adopted for use in the capture, management, and transmission of personal health data [51-54]. These smart devices are outfitted with sensors such as GPS trackers, accelerometers, cameras, and depth sensors and are progressively updated each year with newer and more powerful components. The incorporation of these advanced sensors may offer a scalable solution for population-wide telemonitoring of nutritional status and allow early detection of malnutrition in the community.

### Facial Morphometrics and Malnutrition Screening

A relatively unexplored method that may offer predictive capabilities in the area of malnutrition screening is the application of facial morphometrics. Facial morphometrics is the study of the contours and structures of faces using geometric mapping tools that allow landmarks and features to be identified [55]. Although facial changes during weight loss and undernutrition have been reported since the time of Leonardo Da Vinci, their implications and ability to predict nutritional status have been recognized only recently [56-62].

The newly integrated depth sensors in personal smart devices may present a novel solution that may be beneficial for the remote monitoring of malnutrition. These depth sensors capture 3D depth data of objects, enabling the use of applications such as augmented reality–based applications, as well as the generation of 3D point clouds for 3D modeling [63]. Most recently, a notable application that highlights the current level of sophistication and accuracy of depth sensors is Apple’s 3D face recognition–based encryption system, known as Face ID [64]. The degree of security offered by the Face ID system can be attributed to the level of 3D detail that the True Depth camera can capture. A total of 30,000 infrared dots are projected onto the target object and captured by an infrared-sensitive camera, allowing fine details such as contours and recesses to be detected by the camera [65]. This granularity has enabled its use in a wide range of applications, ranging from the analysis of human facial emotions to the customization of prosthetics and medical equipment requiring precise fit [63,66-71].

With depth cameras becoming the mainstay in smartphones beyond the iPhone, there may be potential for developing telemonitoring applications that involve the extraction and correlation of facial morphological features with health indices. Typical facial landmarks include the eyes, nose, chin, cheekbones, and the overall shape of the face. The extraction and analysis of features such as geodesic distances or ratios between these landmarks can serve as informative discriminatory tools in the quantification of shape and variation [72].

These quantified features could serve as proxy markers for the nutritional status of the human body. This has been demonstrated in several studies, which have observed correlations between superficial tissues that affect the overall morphology of the face such as the zygomatic fat pads and masseter and temporalis muscles with nutritional status and whole-body SMM [73-83]. The quantification of these tissues involves the use of techniques such as ultrasound, MRI, and CT scans. However, these methods can be expensive, time consuming, and stressful for patients. Therefore, the application of facial morphometrics using widely available depth sensors could provide a more accessible and less invasive solution for larger populations.

The use of facial morphometrics in the field of health sciences is not new. However, most of the research has primarily been conducted in the fields of aesthetics, orthodontics, and prosthetics. There have been attempts to explore the correlation between facial features and nutrition-focused indices such as BMI and waist-to-hip ratio [84-91]. Earlier studies used 2D images and relied on planar distances or 2D angularity to identify features that account for BMI variations [87,89,92-99]. Recent studies have explored the use of 3D meshes for more detailed geometric modeling of faces using tools such as 3D scanners or stereophotogrammetry [86,100-102]. However, these technologies require a specialized setup, and individuals must be physically present for the scans to be conducted, thus limiting the number of individuals that can be recruited. The widespread availability of personal smartphones integrated with accurate depth cameras presents an opportunity for more scalable research in this area, allowing the acquisition of larger data sets within a shorter amount of time.

With these capabilities in mind, this paper further discusses the relevance of certain facial features that have the potential to provide diagnostic value in identifying changes in SMM and overall nutritional status.

## Facial Features With Clinical Significance

### Overview

With imaging tools capable of deriving facial morphology accurately, both direct and indirect tissue losses can potentially be captured as additional features for analysis. Direct measurements of tissue loss can include the quantification of protrusions and depressions that form from tissue atrophy, whereas indirect measurements include features that become more prominent when there is a loss of fat or muscle tissue, such as certain bone processes and ridges, or the angulation of the jawline [73,100,103].

The degree of bone prominence is important in determining nutritional status [104,105]. Physical examinations of malnutrition typically observe the prominence of bone structures in the clavicle, scapular, acromion, and patellar regions to determine the degree of muscle and subcutaneous fat loss [104]. Certain superficial structures that have a lesser degree of covering tissue, such as the zygomatic process or mandible, are immediately distinguishable. Other bone structures such as those in the orbit, zygomatic bone, and other anatomical details of the mandible or jaw may become more prominent in the case of malnourishment, following the loss of muscle and fat tissue [105].

### Segmentation of the Face

**Figure 1:**
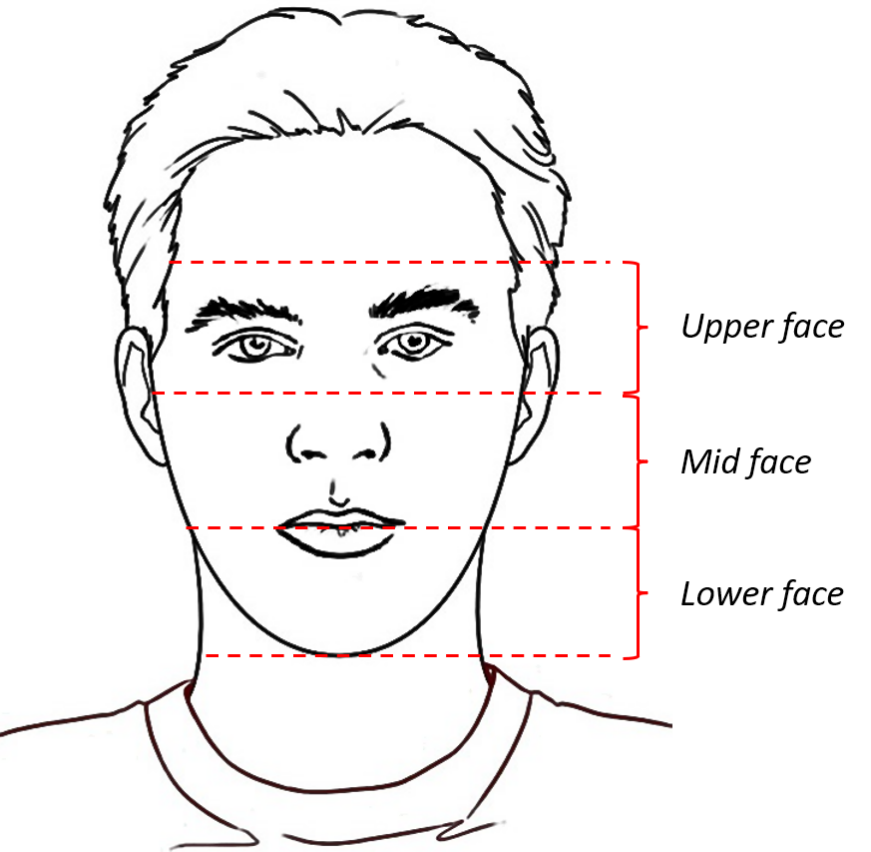
Illustration of face segments of interest.

To discuss potential features, we segment the face into three regions: upper face, midface, and lower face (Figure 1).

#### Upper Face

The upper face comprises the regions between both temples, including the eyes and the surrounding periorbital region. The primary superficial tissues that contribute to craniofacial morphology in this region include the temporalis muscle, periorbital fat pads, and bones that make up the orbital rim [59,106,107]. These features are significantly affected by malnutrition and loss of fat or skeletal muscle in the region [104,108].

The temporalis muscle, as well as the superficial and deep temporal fat pads, contribute to most of the volume in the temple regions [109]. As it is easily accessible and palpable, the temporalis muscle is frequently used as an indicator of malnutrition in physical examinations [104]. The temporalis muscle is a fan-shaped muscle situated beneath the temples, which runs from the side of the skull down to the back of the lower jaw, forming a convexly shaped area that blends in with the lateral orbital rim [107]. On several occasions, this muscle has been shown to be an effective surrogate marker for sarcopenia and SMM in the rest of the body. Many studies have reported correlations between the temporalis muscle and other indicators of SMM, such as the psoas muscle, lumbar skeletal muscle cross-sectional area, and patient grip strength [75,76,78,82,83,110-112].

In the case of atrophy of the underlying fat pads and the temporalis muscle, this region could appear sunken, thus increasing the prominence of the temporal orbital rim [106]. The frontal process of the zygomatic bone and the superior border of the zygomatic arch become more prominent and can result in sharp angulation along the orbital rim [109]. These features cast a shadow that accents the temporal hollows, resulting in a gaunt appearance [106].

**Figure 2:**
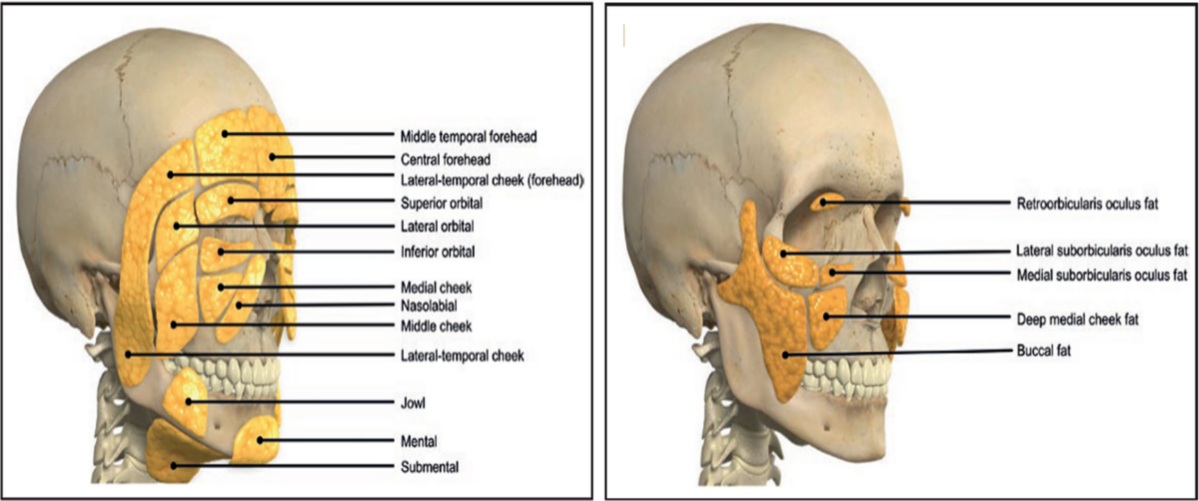
Overview of facial fat pads. Superficial fat compartments in the midface include the nasolabial fat pad, medial cheek, buccal fat pad, and deep medial cheek fat (Swift et al [115]).

The morphology of the periorbital region also changes significantly in the case of tissue loss and is largely determined by the volume of the periorbital fat pads. These comprise the temporal fat pad, the preaponeurotic fat pad, the nasal fat pads, and the central fat pad [113] (Figure 2). In cases of lipoatrophy, the loss of volume in these fat pads has been observed to accentuate the superior, medial, and lateral edges of the orbital rim, contributing to a sunken, skeletonized appearance [109]. The increasing hollowness of the orbit coupled with skin laxity can also contribute to the development of infraorbital dark circles [114].

Certain confounders may arise because of the natural changes around the eye regions, which may complicate the identification of malnutrition. Age contributes to a decrease in brow projection from the superior orbital rim [106]. Youthful brows have a more prominent anterior projection because of the underlying support from the retro-orbicularis oculi fat. During aging, subsequent remodeling and thinning of the superomedial and inferolateral orbital rim can result in changes to the morphology and decreased projection of the brows from the superior orbital rim [106]. This makes the superciliary arch more prominent and visible in older adults. Therefore, it is important to carefully distinguish this from excess atrophy of fat and muscle tissue.

It has been well established that the morphology around the periorbital fat pads is affected by many factors because of aging [59,116,117]. The tissues around the eyes, such as the muscles that support the eyelids, tend to weaken. Fat pads that support the eye may migrate lower to take the form of bags, and a reduction in fat and underlying muscle fibers can also give the eyelids a sunken and hollow appearance. The loss of skin elasticity and development of eyelid folds or ptosis can lead to additional contours forming around the eye [59]. Future research will need to account for these variabilities that may exist because of age, gender, and ethnicity to differentiate between malnutrition and the natural process of aging [72].

#### Midface

The midface comprises the regions bound by the cheekbones as the upper boundary and the lips as the lower boundary. Key tissue groups that affect the morphology of this region include the masseter muscle, the various zygomatic-malar fat pads, and the zygomatic bone [106,107].

The masseter muscle is a masticatory muscle located over the zygomatic bone, running from the temporal bone to the lower jaw [107]. Given the prominence and superficiality of the masseter muscle on the side of the face, it contributes to the facial contour of the cheek. The thickness of the masseter muscle significantly affects the overall morphology of the face [107,118-128]. The morphological effects of masseter muscle thickness have been well documented in the field of aesthetic medicine, where prescriptive volume loss through the use of botulinum toxin type A demonstrates noticeable changes in the shape of the face [118]. Given the superficiality of the muscle, Murakami et al [77] developed a tool to estimate the mass of the masseter muscle using surface measurements obtained with a vernier caliper. These were paired with thickness measurements derived from ultrasound images to determine the overall mass of the masseter muscle. This was shown to be an accurate method of assessment when compared with the results measured using MRI [77].

Similar to the usability of the temporalis muscle as a surrogate marker for SMM, the masseter muscle has also been found to be correlated with whole-body SMM and the psoas, limb, and trunk muscles [74,77,79,80,126,129]. Relationships have been established between the masseter muscle and aspects of physical fitness in older adults [130]. In some studies, the thickness of the masseter muscle was found to be a better predictor of mortality and sarcopenia than the psoas muscle, which is a commonly referenced muscle in the diagnosis of sarcopenia [78-81,110,131,132].

Given the masseter muscle’s key function as one of the primary muscles of mastication, its strength has also been found to be correlated with the chewing ability [74,130,133]. Research has shown that the development or attrition of the masticatory muscles is related to chewing activity and is indirectly linked to dietary habits [74,77,122,123,130]. In patients who experience loss of appetite or decreased food intake because of medical, social, or psychological reasons, the masticatory muscles have been found to rescind in size [77,81,134]. This will likely affect the external morphology of the cheeks and jaw and could potentially indicate a risk of malnutrition.

**Figure 3:**
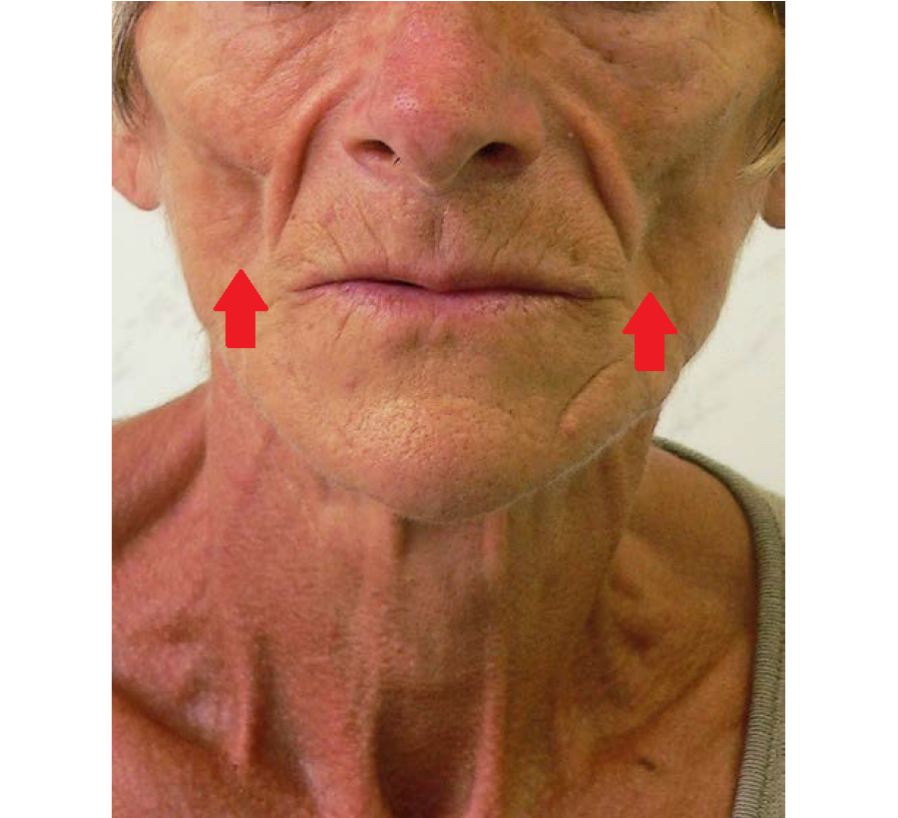
Visible atrophy of adipose tissue and protruding facial bone structures in both cheeks because of antiretroviral therapy for HIV, indicated by red arrows (Szczerkowska-Dobosz et al [105]).

There are several fat compartments in the zygomatic-malar region of the face. The deep midface compartments include the medial and lateral suborbicularis oculi fat, the medial portion of the buccal fat pad, and the deep medial cheek fat. Superficial compartments include the nasolabial and middle cheek superficial fat compartments (Figure 2) [107,116]. The fat pads in this region are typically considered in malnutrition-specific physical examinations. In these examinations, practitioners typically look for hollow, sunken, and narrow faces as these can be an indication of malnutrition [104,108]. In extreme scenarios of tissue loss such as HIV-specific lipoatrophy, exacerbated atrophy in these fat pads can manifest as significant concavities in the buccal and malar areas [105] (Figure 3). This greatly accentuates the zygomatic bone and can form many depressions in the midfacial region.

Subcutaneous buccal fat pads are also a significant area of aging-associated fat loss [59]. The loss of overall volume, decreased tissue support for facial features, and a reduction in skin elasticity can lead to the hollowing of the cheeks. These factors can affect the morphology of the face and, as such, will need to be accounted for when identifying features correlated with malnutrition.

#### Lower Face

The lower face comprises the mandible and the surrounding tissues of the lower cheeks, chin, and neck. These structures contribute significantly to the overall shape of the face and are often easily perceptible, except in cases of excess facial hair. As such, the lower face is a key point of reference in many facial morphological studies and BMI prediction algorithms [89,95,98,135,136].

The lower mandible contributes to the morphology of the lower face and accentuates the jawline. Aesthetic surgeons refer to a youthful jawline as one characterized by a straight line from the chin to the mandibular angle [137,138]. Excess subcutaneous fat in the region may cause certain skinfolds and disrupt this angular definition of the jawline, which can lead to the lower face becoming more squarish than oval [84,89]. Conversely, malnutrition and periods of unintentional weight loss can lead to atrophy of subcutaneous fat and the underlying muscle tissues, resulting in a more pronounced jawline.

**Figure 4:**
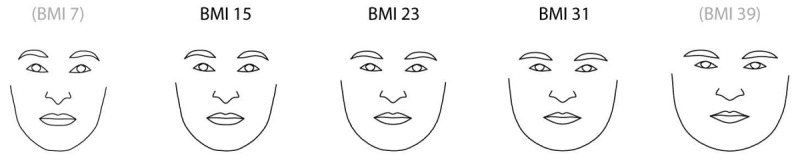
Changes in BMI (kg/m2) as reflected in face shape (Mayer et al [89]).

**Figure 5:**
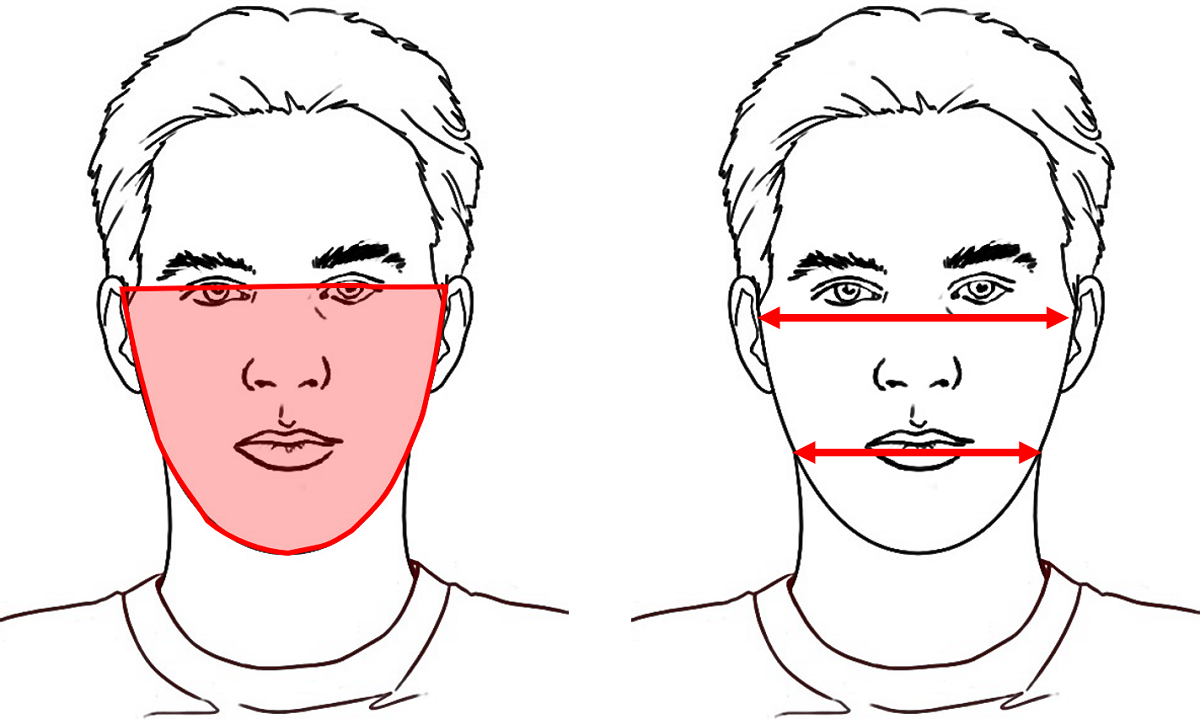
Perimeter-to-area ratio (left) and cheek-to-jaw width ratio (right; adapted from Coetzee et al [84]).

The jawline can be a potentially important feature in predicting malnutrition or sarcopenia. In a study exploring the relationship between face shape and waist-to-hip ratio, Mayer et al [89] showed that a 2D face shape had a correlation coefficient of 0.63 (Figure 4). Participants with a lower BMI were likely to have jawlines that were sharper and more angular, whereas the jawlines of participants with a higher BMI were wider and rounder. In the Coetzee et al [84] study identifying quantifiable visual cues that contribute to weight, 2 out of the 3 primary cues that showed predictive potential to determine BMI from facial images were the perimeter-to-area ratio and the cheek-to-jaw width ratio (Figure 5). These features are largely dependent on the amount of tissue in the lower facial regions, thus indicating the importance of identifying the jawline as a differential landmark.

Although subcutaneous fat affects the overall shape of the jawline, aging is another important factor that changes the overall morphology of this facial feature [106,135,138-140]. A strong mandibular border with prominent jawline definitions is typically a sign of a youthful face, whereas aging contributes to squaring of the face and a loss of jawline definition. This is because of the descent of the hyoid bone and larynx, leading to a blunting of the cervicomental angle. Together with ptosis of the unsupported skin, chin pad, and facial portion of the platysma muscle, it results in the development of ptotic jowls [106,135,141]. This can potentially complicate the analysis of mandibular morphology and can affect its usability in malnutrition assessment, especially in older adults. Other confounding factors that may mask the contours of the chin and mandible regions include facial hair or aesthetic fillers.

## Challenges

Facial morphology technology provides several exciting opportunities as a diagnostic tool for malnutrition and body composition. However, there remain important challenges that need to be overcome.

Developing a comprehensive database that accounts for potential confounders and isolating the degree of correlation between various facial morphological features and overall SMM and nutritional status is necessary. Many factors can influence facial variations outside of weight and SMM, such as gender, ethnicity, age, and medical conditions [72,142,143]. The rate and degree of facial development throughout the course of growth spurts and aging may also vary depending on ethnicity, which could further complicate the effects of either parameter [144]. The development of facial morphology databases will need to account for these factors such that specific features most relevant to malnutrition can be isolated.

Facial morphology holds potential as a diagnostic tool in the context of malnutrition on the premise that SMM is correlated with facial tissues. However, this correlation may not always be consistent, particularly for complex medical conditions. Certain diseases such as critical illness myopathy have been noted to affect muscles in the body differently, resulting in significant muscle loss in the limbs and trunk while sparing the masseter and other craniofacial muscles [142]. Controlling for these potential confounders will become an important consideration if facial morphology is to be used as a population-wide diagnostic tool.

There can also be disproportionate tissue loss in specific parts of the face, which may confound the degree of importance of the respective facial regions. For example, patients undergoing highly active antiretroviral therapy for HIV infections or patients with connective tissue diseases such as panniculitis are known to develop asymmetrical and dynamic facial lipoatrophy [105]. This form of lipoatrophy can manifest in different ways in age- or lifestyle-related malnutrition, and care should be taken to distinguish between the two.

The use of facial morphology as a diagnostic tool may also require additional considerations because of aesthetic variables. The presence of facial hair, such as beards and mustaches, may impair the ability of features derived from the lower half of the face to be used in the analysis. Cosmetic or reconstructive surgery used for the concealment of facial anomalies or to improve one’s appearance is also becoming relatively common in many countries [145]. These can have a significant impact on the morphology of the face and are important factors to consider in these demographics.

Certain ethical and societal considerations may need to be reviewed to increase the adoption of facial morphometrics as a tool in public health. The face is an important and sensitive identifiable feature, and any application incorporating digital 3D scans of the face will need to ensure that patient privacy and data security are not compromised. The development of digital health applications using personal smartphones may also widen the digital divide and potentially alienate certain segments of the population [146]. Populations of lower socioeconomic status may not have access to these devices, and older adult patients may have difficulties navigating certain aspects of newer technologies [35]. These populations are the most vulnerable to malnutrition and its associated complications. To ensure that these segments of the population who may not be able to afford or be familiar with digital technologies are not excluded, there is a need to consider both affordability as a potential barrier to entry, as well as the ergonomics and accessibility of the user experience.

## Discussion

### Conclusions

Undiagnosed malnutrition is becoming a significant problem that can reduce the quality of life of many individuals and inflate the costs of existing health care systems because of the many metabolic complications it can cause. Many high-income countries are faced with a rapidly aging population that may lead to poor food intake, disease etiology, and improper access to food, which may give rise to an increase in the prevalence of malnutrition. Given the need for specialized equipment and trained practitioners for the diagnosis of malnutrition, the development of simpler and more scalable methods for assessing malnutrition has been a long-standing challenge for public health.

Recent advancements in artificial intelligence, computing, data science, and infocommunication technologies present a wealth of opportunities that will shape and revolutionize the landscape of health care. This paper aims to stimulate further discussion and discourse on how adopting this emerging technology could provide real-time access to nutritional status. We explored the feasibility of a novel, noninvasive approach to tackling malnutrition in the community. The use of facial morphometrics extends the use of currently available technology, providing a scalable and easily deployable method for the real-time monitoring of nutritional status. Clinicians and dietitians will be able to keep track of patients remotely and provide the necessary intervention measures as required. Finally, it will furnish health care institutions and policy makers with essential information that can facilitate necessary interventions targeted at affected populations.

## Abbreviations

CT: computed tomography
MRI: magnetic resonance imaging
SMM: skeletal muscle mass
